# Autosegmentation based on different-sized training datasets of consistently-curated volumes and impact on rectal contours in prostate cancer radiation therapy

**DOI:** 10.1016/j.phro.2022.04.007

**Published:** 2022-05-05

**Authors:** Caroline Elisabeth Olsson, Rahul Suresh, Jarkko Niemelä, Saad Ullah Akram, Alexander Valdman

**Affiliations:** a Medical Radiation Sciences, Clinical Sciences, Sahlgrenska Academy, Gothenburg University, Gothenburg, Sweden; b Regional Cancer Centre West, Western Sweden Healthcare Region, Gothenburg, Sweden; c MVision AI Oy, Helsinki, Finland; d Department of Radiotherapy, Karolinska University Hospital, Stockholm, Sweden

**Keywords:** Radiation therapy, Deep-learning, Autosegmentation, Prostate cancer, Rectum, CT

## Abstract

**Background and purpose:**

Autosegmentation techniques are emerging as time-saving means for radiation therapy (RT) contouring, but the understanding of their performance on different datasets is limited. The aim of this study was to determine agreement between rectal volumes by an existing autosegmentation algorithm and manually-delineated rectal volumes in prostate cancer RT. We also investigated contour quality by different-sized training datasets and consistently-curated volumes for retrained versions of this same algorithm.

**Materials and methods:**

Single-institutional data from 624 prostate cancer patients treated to 50–70 Gy were used. Manually-delineated clinical rectal volumes (*clinical*) and consistently-curated volumes recontoured to one anatomical guideline (*reference*) were compared to autocontoured volumes by a commercial autosegmentation tool based on deep-learning (*v1*; n = 891, multiple-institutional data) and retrained versions using subsets of the curated volumes (*v32/64/128/256;* n = 32/64/128/256). Evaluations included dose-volume histogram metrics, Dice similarity coefficients, and Hausdorff distances; differences between groups were quantified using parametric or non-parametric hypothesis testing.

**Results:**

Volumes by *v1-256* (76–78 cm^3^) were larger than *reference* (75 cm^3^) and *clinical* (76 cm^3^). Mean doses by *v1-256* (24.2–25.2 Gy) were closer to *reference* (24.2 Gy) than to clinical (23.8 Gy). Maximum doses were similar for all volumes (65.7–66.0 Gy). Dice for *v1-256* and *reference* (0.87–0.89) were higher than for *v1-256* and *clinical* (0.86–0.87) with corresponding Hausdorff comparisons including *reference* smaller than comparisons including *clinical* (5–6 mm vs. 7–8 mm).

**Conclusion:**

Using small single-institutional RT datasets with consistently-defined rectal volumes when training autosegmentation algorithms created contours of similar quality as the same algorithm trained on large multi-institutional datasets.

## 1 Introduction

In radiation therapy (RT), contour/volume variability continues to be a problem, in particular for non-tumour tissue or organs at risk (OARs) [1], [2], [3], [4], [5]. Dose fall-off margins in modern RT can be set extremely tight meaning that correct volume definitions are more critical now compared with previous RT delivery techniques. In the clinic, OARs are either manually delineated by RT professionals in treatment planning systems or proposed by autosegmentation tools, primarily based on atlases or artificial intelligence. The latter has emerged over the last decade as a time-saving means for the contouring task but also to reduce intra- and interobserver variations [6], [7], [8], [9], [10], [11]. This, in turn, opens up new possibilities to harmonize OAR volumes and interpretations of associated dose metrics and normal-tissue complication probability risk estimates. Knowledge about how autosegmentation tools perform on different datasets is, however, limited. In addition, it is unknown if “calibration” to standard (benchmark) datasets can increase their performance in the clinic.

Autosegmentation algorithms based on deep learning are typically developed in three steps, which encompass training, validation, and testing [9]. The basis for algorithm development is often curated clinical or research data with diverse patient anatomies since algorithm performance depend on image characteristics included in the training datasets. In practice, datasets used for algorithm development are split so that the majority of images is used for training, some 10–20% for validation, and 10–30% for testing [12], [13], [14]. There are no consensus requirements for how many patient anatomies are needed in either of these datasets to arrive at accurately defined volumes for a particular structure in the clinical setting [9], with agreement between autocontoured and manually-delineated expert volumes typically being quantified using volume overlap metrics such as the Dice similarity coefficient [15]. For training dataset sizes, reported numbers in the current scientific literature range from 50 patients and upwards [12], [13], [14]. Data curation is a time-consuming task making it challenging to obtain large datasets for this purpose. There are also often numerous options for data curation since consensus guidelines on how to define specific OAR volumes for many body regions diverge in the RT community with many clinics using their own established protocols for structure delineations [9].

The aim of this study was to determine agreement between rectal volumes by an existing autosegmentation algorithm based on deep learning and manually-delineated rectal volumes in prostate cancer RT. A specific objective was to investigate contour quality by different-sized training datasets and curated rectal volumes recontoured to the Swedish STRONG OAR guideline for male pelvis [16] for retrained versions of this same algorithm.

## 2 Materials and methods

OAR data investigated in this study were taken from 624 patients treated for prostate cancer at the Sahlgrenska University Hospital, Gothenburg, Sweden, in 2018–2019. All patients had been treated with volumetric-modulated arc therapy (6 MV photon-beam radiation quality) to prescribed doses of 50/66/70 Gy using 2 Gy/3 Gy/2 Gy/fraction, respectively. The majority of patients had been treated to 66 Gy, patients receiving 50 Gy had undergone additional brachytherapy, and patients receiving 70 Gy had undergone surgical removal of the prostate prior to salvage RT. Ethical permit for the study was granted from the Swedish Ethical review authority (No: 641–17, T1115-18, 2020–04108).

All patients had been planned for RT using the treatment planning system Eclipse^TM^ (Varian Medical Systems; version 15.6). For the work described below, the patients were split in two datasets, where one part was used to evaluate performance of the retrained algorithm versions (Dataset 1, n = 299) and the other part was used for algorithm development (Dataset 2, n = 325).

Patient treatment characteristics and the characteristics of the studied rectal OAR volumes and doses are presented in Table 1.

**Table 1:** Patient treatment groups and characteristics of the studied manually-delineated reference and clinical rectal volumes and doses in the different datasets used for testing (Dataset 1) and retraining (Dataset 2) of the MVision autosegmentation algorithm (Eclipse^TM^ original data).

	Study group:	Reference	Clinical	TD=50 Gy	TD=66 Gy	TD=70 Gy	TD=Other
Dataset (*No*.)	Metric\Unit:	cm^3^/Gy	cm^3^/Gy	*n (%)*	*n (%)*	*n (%)*	*n (%)*
ALL (*n=624*)	Volume	72.3±26.8	73.4±26.7	118	295	197	14
	Mean dose	24.4±6.3	24.0±5.8	(19)	(47)	(32)	(2)
	Max. dose	65.9±7.8	65.9±7.9				
Dataset 1 (*n=299*)	Volume	73.9±29.8	75.5±29.9	50	167	75	7
	Mean dose	24.5±6.3	24.0±5.8	(17)	(56)	(25)	(2)
	Max. dose	65.9±8.0	65.8±8.2				
Dataset 2 (*n=325*)	Volume	70.9±23.6	71.5±23.3	68	128	122	7
	Mean dose	24.2±6.3	24.0±5.9	(21)	(39)	(38)	(2)
	Max. dose	65.9±7.7	65.9±7.7				
*training* (*n=32*)	Volume	72.1±28.9	72.5±25.8	7	14	11	0
	Mean dose	24.3±6.5	23.6±5.2	(22)	(44)	(34)	(0)
	Max. dose	65.8±7.7	72.3±7.7				
*training* (*n=64*)	Volume	70.4±26.1	70.4±24.8	12	24	26	2
	Mean dose	25.1±6.3	24.8±5.6	(19)	(38)	(41)	(3)
	Max. dose	66.3±7.5	66.3±7.5				
*training* (*n=128*)	Volume	68.5±23.5	69.8±23.1	22	55	48	3
	Mean dose	24.9±6.1	24.3±5.4	(17)	(43)	(38)	(2)
	Max. dose	66.4±7.2	66.4±7.2				
*training* (*n=256)*	Volume	69.4±22.9	70.6±22.5	55	98	99	4
	Mean dose	24.5±6.5	24.0±5.9	(22)	(38)	(39)	(1)
	Max. dose	65.9±7.8	65.9±7.8				

Abbreviations: Gy = Gray, Max. = maximum, No.= number, TD = total (prescribed) dose to treatment region.

Note that *reference* volumes were somewhat smaller than *clinical* volumes with somewhat higher mean doses but similar maximum doses when comparing all patients. Volumes and dose metrics in Dataset 1 were comparable with corresponding metrics in Dataset 2 as were characteristics of the four sub-cohorts from Dataset 2 to the overall characteristics of Dataset 2.

### 2.1 Organs at risk and related data

For all patients, rectum had been manually contoured as an OAR in clinical routine on planning CT images used for treatment. Images had been required at a resolution of 512 by 512 pixels with 2 mm slice thickness, typically with a voxel size of 1.074x1.074x2 mm^3^. As an overall principle, clinical rectal volumes had been defined by their outer contours, 5 cm in the cranial direction from the centre of the prostate and down to the anal verge in the caudal direction (uncurated volumes, referred to as *clinical*). Rectum was also consistently recontoured for all patients in the research version of EclipseTM according to the STRONG OAR guideline [16], in line with international consensus recommendations [17], [18]. This meant that rectum was defined by its outer contour with the cranial border starting at the point when rectum loses its round shape in the axial plane and connects anteriorly with the sigmoid and the caudal border ending at the lowest level of the ischial tuberosities (curated volumes, referred to as *reference*). To avoid interobserver variability, manual recontouring was done by one project member with the aid of a contouring manual and under supervision from a senior oncologist (AV).

DICOM-RT data (CT images and structure set files) including clinical and recontoured rectal volumes were extracted for all patients in anonymized format from Eclipse^TM^ to provide detailed volume and dose information.

### 2.2 Autosegmentation

The commercial version of the MVision algorithm for autocontouring was initially used to automatically identify rectal volumes in Dataset 1 (original algorithm version 1.2.1, referred to as *MVision_v1*; MVision Segmentation Service, Mvision AI Oy Helsinki, Finland). The MVision prediction model for male pelvis was also retrained to create four new versions with training subsets from Dataset 2 including curated rectal volumes for 32, 64, 128, and 256 patients (retrained algorithm versions, referred to as *MVision_v32*, *MVision_v64*, *MVision_v128*, and *MVision_v256*, respectively). Validation of training was done using a different subset from Dataset 2, randomly selected but not used for the training part. Small-scale testing of performance was done using a third subset from Dataset 2, also randomly selected but not used neither for the training part nor for the validation part. Large-scale testing of performance was finally done using Dataset 1.

**Fig. 1:**
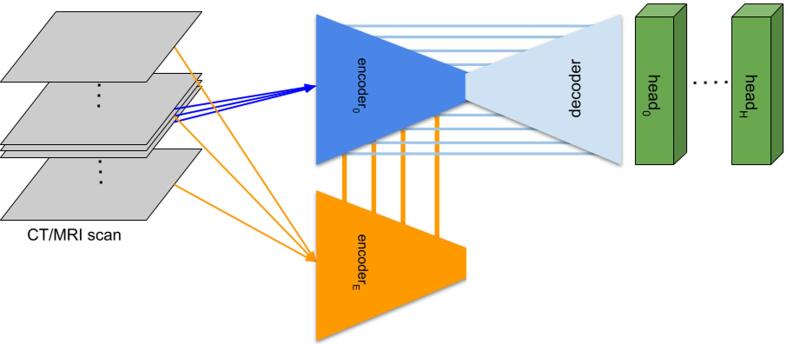
The model architecture of the MVision deep-learning algorithm. The input of the network are thin sections of the 3D scan. There are multiple encoders, each taking three slices as input. The n-th encoder takes slices [-(n + 1), 0, (n + 1)] as its input. The feature maps of the n-th global encoder are summed with the feature maps of the (n-1)-th encoder. There is a single decoder (light blue), which receives 4 features maps from the 0-th (local) encoder. There are H segmentation heads (green), with each predicting masks of multiple non-overlapping regions of interest. Abbreviations: CT = computed tomography; MRI = magnetic resonance imaging. (For interpretation of the references to colour in this figure legend, the reader is referred to the web version of this article.)

Details on the original algorithm version including model architecture are presented in Fig. 1 and in the Supplementary material together with outputs from the four new algorithms during retraining.

### 2.3 Statistics

The degree of variation between rectal volumes was investigated using the Dice similarity coefficient for volume overlap and the Hausdorff distance for maximum distance between volumes. For comparisons involving these metrics and the autocontoured volumes, outputs from *MVision_v256* were used as reference. Differences were also quantified using volume and dose metrics (mean and maximum dose) as provided by dose-volume histograms (DVHs) in CERR [19]. Comparisons between groups were made using the paired Student’s *t*-test (when assuming dependency between samples, i.e. within-patient comparisons) and the Wilcoxon rank sum test or the Student’s *t*-test (when assuming independency between samples, i.e. between-patient comparisons), depending on the distribution of the underlying data. Two-sided p-values ≤ 0.05 were assumed to indicate statistically significant differences. No adjustments for multiple comparisons were made. Results are reported as mean ± standard deviation (SD) and median (range), whichever most applicable. Statistical analyses, including image- and dose data processing, were performed in MATLAB’s statistics and image toolboxes (Math-Works, Natick, MA, USA) and using the Python programming language (primarily the PyTorch package [20]).

## 3 Results

### 3.1 Performance of the original MVision algorithm version

**Fig. 2:**
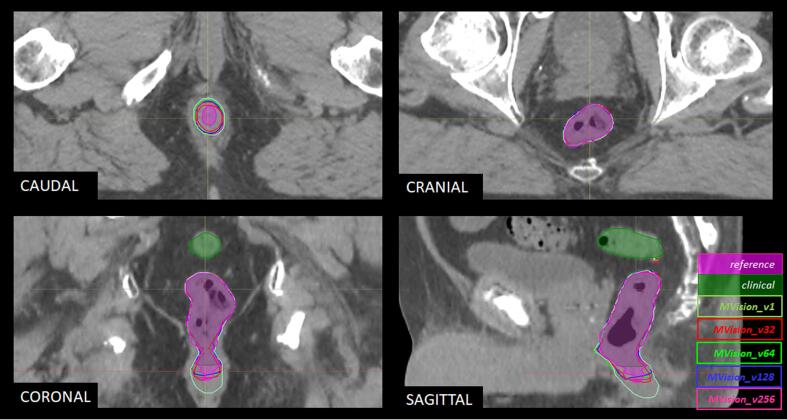
Example outputs from original and retrained versions of the MVision deep-learning algorithm on planning-CT images for one prostate cancer case. Note how the original version of the autosegmentation algorithm (MVision_v1; colour coding in image) results in a volume similar to the reference curated volume (filled purple) in the cranial direction but below the reference in the caudal direction and with a somewhat larger circumference. The recalibrated versions (MVision_v32-256; colour coding in image) remain at the same anatomical border as the original version but approach the lower anatomical border and the circumference of the reference. The anatomical border of the clinical uncurated volume (filled green) is above the reference in the cranial direction. (For interpretation of the references to colour in this figure legend, the reader is referred to the web version of this article.)

*MVision_v1* produced volumes of 78.0 ± 28.7 cm^3^, numerically closer to *clinical* (p = 0.09) than to *reference* (p = 0.010) (Table 2, Fig. 2). Mean doses for *MVision_v1* (25.2 ± 6.3 Gy) were statistically significantly higher than mean doses of both manually-delineated volumes, although, numerically closer to *reference* (p = 0.017) than to *clinical* (p < 0.001) whilst no difference in maximum doses between *MVision_v1* (66.0 ± 8.1 Gy) and either volume representation could be determined (p > 0.05 for both comparisons).

**Table 2:** Characteristics and comparisons between manually-delineated uncurated (clinical) and curated (reference), as well as auto-contoured rectal volumes and doses in 299 patients used for large-scale testing of the retrained versions of the MVision autosegmentation algorithm (MVision data).

n=299		*MVision*				
Metric/	Algorithm	*v1*	*v32*	*v64*	*v128*	*v256*
OAR volume	version:	(p-value)	(p-value)	(p-value)	(p-value)	(p-value)
**Volume**	*mean±SD (cm^3^)*	*78.0±28.7*	*75.7±30.8*	*77.2±30.5*	*77.1±31.3*	*77.5±30.7*
Reference	*74.7±30.0*	(0.010)	(0.021)	(<0.001)	(<0.001)	(<0.001)
Clinical	*76.4±30.1*	(0.092)	(0.817)	(0.038)	(0.053)	(0.015)
**Mean dose**	*mean±SD (Gy)*	*25.2±6.3*	*24.4±6.5*	*24.2±6.4*	*24.2±6.3*	*24.2±6.3*
Reference	*24.4±6.3*	(0.017)	(0.637)	(0.079)	(0.076)	(0.028)
Clinical	*23.8±5.7*	(<0.001)	(0.009)	(0.104)	(0.089)	(0.146)
**Max. dose**	*mean±SD (Gy)*	*66.0±8.1*	*65.8±8.2*	*65.7±8.1*	*65.8±8.2*	*65.9±8.1*
Reference	*65.7±7.9*	(0.829)	(0.328)	(0.745)	(0.635)	(0.631)
Clinical	*65.7±8.1*	(0.318)	(0.719)	(0.141)	(0.605)	(0.179)

Abbreviations: Gy = Gray, Max. = maximum, OAR = organ at risk, SD = standard deviation.

The Dice values for volume overlap between *MVision_v1* and *clinical* were numerically similar to Dice-values for volume overlap between *MVision_v1* and *reference* (mean = 0.86–0.87 with SD: 0.05–0-06; Table 3), yet favoring *reference*. The Hausdorff values for maximum distance between volumes were larger for comparisons between *MVision_v1* and *clinical* than for comparisons between *MVision_v1* and *reference* (7.0 ± 6.1 mm vs. 4.8 ± 3.7 mm), also favoring *reference*.

**Table 3:** Volume overlap (Dice), maximum distance (Hausdorff) between volumes and algorithm performance between auto-contoured rectal volumes by different versions of the MVision algorithm and manually-delineated uncurated (clinical) and curated (reference) rectal volumes (MVision data).

OAR volume	Reference	Reference	Clinical	Clinical	Reference vs. Clinical
Algorithm	Dice	p-value	Dice	p-value	Dice (p-value)
Mvision_v1	0.87±0.05	<0.001	0.86±0.06	0.465	(0.001)
MVision_v32	0.86±0.07	<0.001	0.85±0.07	<0.001	(<0.001)
MVision_v64	0.87±0.07	<0.001	0.86±0.07	0.002	(<0.001)
MVision_v128	0.88±0.07	0.004	0.86±0.07	0.087	(<0.001)
MVision_v256	0.89±0.07	*ref.*	0.87±0.07	*ref.*	(<0.001)

**Algorithm**	**Hausdorff**	**p-value**	**Hausdorff**	**p-value**	**Hausdorff (p-value)**

MVision_v1	4.9±3.7	0.539	7.0±6.1	0.300	(<0.001)
MVision_v32	6.1±5.6	<0.001	7.9±6.2	0.066	(<0.001)
MVision_v64	5.4±5.3	0.017	7.5±6.2	0.483	(<0.001)
MVision_v128	5.3±5.3	0.010	7.2±6.0	0.578	(<0.001)
MVision_v256	4.7±5.3	*ref.*	7.3±6.7	*ref.*	(<0.001)

Note that comparisons between algorithm versions are made row-wise with Mvision_v256 as reference (in leftmost columns), and comparisons in performance between a same algorithm version and manually-delineated volumes are made column-wise (rightmost column).

Abbreviation: OAR = organ at risk; ref.=reference.

### 3.2 Performance of the retrained MVision algorithm versions

**Fig. 3:**
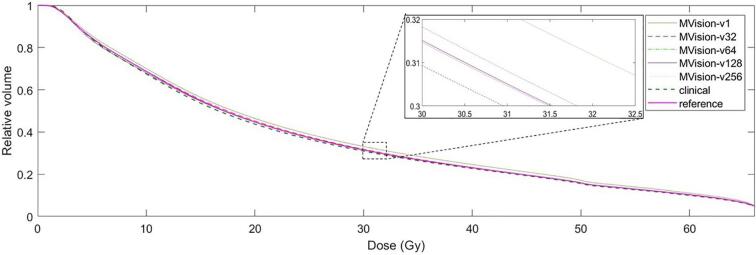
Averaged DVHs of original (MVision_v1) and retrained versions (MVision_v32/64/128/256) of the MVision deep-learning algorithm for autocontouring of rectum. Numbering for retrained versions indicates training dataset size during algorithm development.

Volumes by the four new versions of the MVision algorithm were typically somewhat larger than both manually-delineated volumes. *MVision_v32* produced the smaller volumes (75.7 ± 30.8 cm^3^) and *MVision_v256* the larger (77.5 ± 30.7 cm^3^) with the smallest volume closer to *clinical* (p > 0.05) than to *reference* (p = 0.021; Table 2, Fig. 2). Mean doses for the new versions were in between the mean doses of *clinical* and *reference*, 24.2 ± 6.3 Gy (*MVision_v256*) to 24.4 ± 6.5 Gy (*MVision_v32*), with the highest mean dose closer to the mean dose of *reference* (p > 0.05) than to the mean dose of *clinical* (p = 0.009). There were no differences between maximum doses for any autosegmented volume and either manually-delineated volume (65.7–65.9 Gy with SD: 8.1–8.2 Gy; p > 0.05 for all comparisons). On a DVH level, differences in volumes and doses were marginal (Fig. 3). Where differences could be noted, the same relative volume of the *reference* DVH and the *MVision_v32* DVH typically related to a higher dose than of the *clinical* DVH and the DVHs of the other retrained algorithm versions.

The Dice values for volume overlap ranged from 0.85 ± 0.07 (*MVision_v32*) to 0.87 ± 0.07 (*MVision_256*) with respect to *clinical* and from 0.86 ± 0.07 (*MVision_v32*) to 0.89 ± 0.07 (*MVision_256*) with respect to *reference* (Table 3). With respect to comparisons between algorithm versions, and with the Dice values of *MVision_v256* as reference, differences in Dice were noted for comparisons involving *clinical* and *MVision_v32/64* (p ≤ 0.002). For corresponding comparisons involving *reference*, differences in Dice were noted for all three comparisons (p ≤ 0.004). Performance of any algorithm version with respect to volume overlap was in favor of comparisons including *reference.*

The Hausdorff values for maximum distance between volumes ranged from 7.2 ± 6.0 mm (*MVision_v128*) to 7.9 ± 6.2 mm (MVision_v32) with respect to *clinical* and from 4.7 ± 5.3 mm (*MVision_v256*) to 6.1 ± 5.6 mm (*MVision_v32)* with respect to *reference* (Table 3). With respect to similar comparisons between algorithm versions as for the Dice values, but with the Hausdorff values of MVision_v256 as reference, differences in Hausdorff values were not noted for comparisons involving *clinical* (p > 0.05 for all). In contrast, differences in performance were noted for all comparisons involving *reference* (p ≤ 0.017). Performance of any algorithm version with respect to maximum distance between volumes was in favor of comparisons involving *reference.*

## 4 Discussion

We found that volume overlap between manually-delineated and autosegmented rectal volumes by retrained versions of a commercial deep-learning autosegmentation algorithm (trained on ≤ 30% of originally-used number of patients) was comparable to the corresponding output as created by the original algorithm version. Increased volume agreement by the retrained algorithm versions was more notable for comparisons involving the consistently-recontoured volumes than for comparisons involving the uncurated clinical volumes. Both original and retrained algorithm versions were numerically closer to the clinical volumes whilst mean doses were numerically closer to the recontoured volumes. Maximum doses for both manually-delineated and autosegmented volumes were comparable regardless of algorithm version. The impact of the identified differences between algorithm versions was small on a DVH level.

Performance of any autosegmentation algorithm depends on the underlying training dataset quality. The training approaches based on large multi-institutional real-life clinical datasets inevitably suffer from inconsistencies in structure definitions. In contrast, relatively small well-controlled datasets that are consistent with a reference definition may represent a better basis for the training of deep-learning autosegmentation algorithms. Here we found that training datasets with consistently-curated rectal volumes from 64 patients arrived at comparable results to the original algorithm version, which was based on data from 891 patients (Dice = 0.87/0.86 with respect to curated/uncurated volumes). Of note, training data for the original algorithm were curated by multiple annotators and to a different caudal anatomical landmark than the one specified for our recontoured volumes (inclusion of the anal canal versus lowest level of the ischial tuberosities). This possibly explains why we found that the maximum distance between volumes were smaller for comparisons involving the curated volumes (variation expected caudally only) than for comparisons involving the uncurated volumes (variation expected both cranially and caudally), and why the original algorithm version typically fared similarly to the retrained versions in most situations (Hausdorff = 4.7–6.3/7.0–7.9 mm for curated/uncurated volumes). The quality of the autosegmented volumes only improved to a certain point as the size of the training dataset increased above this number. In comparison to other deep-learning autosegmentation algorithms based on CT-imaging and expert volumes, the higher rectal volume overlap values are reported to be Dice = 0.8–0.9 [11], [12], [13]. Two of these three studies used training datasets in the same range as we investigated here (50 patients [13] and 110 patients [12]), but with unknown basis for data curation and not including large dataset evaluations. The third study recently reported results for another commercial deep-learning autosegmentation algorithm, Limbus Contour build 1.0.22, evaluated on an independent dataset with 50 prostate cancer patients. In this study, rectal volumes were defined according to the clinical judgement of radiation oncologists, similarly as for our clinical volumes [11]. Their investigated algorithm was trained on publicly available data with an average of 328 scans per organ model. It is interesting to note that they found that consistently-defined expert volumes by one radiation oncologist as basis for training resulted in as comparable agreement between autosegmented volumes and expert rectal volumes as inter-observer variability between these same experts (n = 3). Altogether, our results further strengthen the fact that agreement between autosegmented volumes and “ground truth” volumes will be influenced by the data curation quality and that the size of training datasets during algorithm development can be surprisingly small and still produce volumes in line with specific anatomical criteria. Reusing the abovementioned rectal overlap value of Dice = 0.8 as a threshold for clinically acceptable performance of autocontours, a final remark is that the original algorithm version we investigated produced volumes below this threshold in 8% of patients if curated volumes were taken as ground truth and in 10% of patients if uncurated volumes were taken as ground truth. Corresponding numbers for the retrained versions were 6–14% of patients (to curated volumes) and 9–16% of patients (to uncurated volumes), with the lower percentages for larger training datasets (data not shown).

In RT, differences in OAR volumes do not always translate into large dose differences [9], [21] although compliance to trial protocols have been reported to be compromised due to incorrect delineations [2]. We found in our data that autosegmented rectal volumes, irrespective of algorithm version, were overall closer to the manually-delineated uncurated clinical rectal volumes (Δ≈0.5–1 cm^3^) than to the manually-delineated curated rectal volumes (Δ≈1-3 cm^3^), with the larger differences typically generated by the smaller-sized training datasets. Differences in dose were typically smaller for comparisons involving the curated volumes than the clinical volumes and were, on a group level, numerically small (mean dose < 1.5 Gy and maximum dose < 0.5 Gy). Of note, the largest differences for individual cases presented with mean dose differences up to 10 Gy and maximum dose differences up to 5 Gy (data not shown), which underlines the importance of inspection and editing before autocontoured structures are used for treatment planning and delivery. We also found that rectal DVHs were similarly shaped on a group level with the volumes generated from the smallest training dataset marginally shifted towards higher dose. In general, overestimated dose for OARs brings the dose distributions to the safer side, however, underdosage of tumours may be a consequence if the OAR is directly adjacent to the planning target volume and unintendedly included in the high-dose region on the basis of an incorrectly-defined volume [18]. Furthermore, variations in rectum volumes and the impact on rectal toxicity modelling has been investigated in a retrospective study where the same rectum definition as used for our curated volumes was included as one of 13 investigated OAR definitions [21]. This study found that the more distinct differences in DVH shapes between the OAR definitions related to volumes of 60–110 cm^3^, mean doses of 35–50 Gy, and maximum doses of 70–73 Gy (n = 163; 3D-CRT primary and salvage prostate cancer treatments; prescribed dose = 67–76 Gy). Interestingly, they found that the different OAR definitions had no impact on predictive ability of toxicity by the investigated models and DVH metrics. Although their differences originate from an earlier treatment era, it is still unlikely that the small dose differences we found by different algorithm versions in our study, in particularly for maximum dose, which is known to be critical for rectal toxicity [22], would be of importance for predictions of rectal toxicity in the clinic using current prediction models.

The main strength of this study was that we used a large dataset for algorithm retraining and evaluated algorithm performance on two manually-delineated large datasets (n = 299), one curated with OARs recontoured to the same anatomical boundaries and one uncurated with OARs contoured in clinical practice. Investigated anatomies were taken as an unselected sample from the majority of prostate cancer patients treated with modern RT during a one-year period at one of Sweden’s largest RT departments (catchment area ≈2 million inhabitants). The main limitation was that the recontouring of rectum in practice resulted in changed anatomical borders with respect to clinical volumes in the cranial and caudal directions only. Therefore, the reported results primarily reflected these geometric variations. This also explains why maximum dose was almost the same on a group level for both autosegmented and manually-delineated volumes with higher doses typically found in the central parts of the rectum, which were included in all investigated volumes. Finally, our results were based on data for one specific pelvic OAR with a reasonably well-defined geometry. Whether they apply to OARs of more complex geometries needs to be further studied.

In conclusion, a relatively small but well-curated dataset with consistently-defined anatomical boundaries for the training of deep-learning autosegmentation algorithms has the potential to create structure volumes and doses of acceptable quality for clinical use. The investigated algorithm, in both its original version and retrained versions, provided reasonable-quality rectal volumes in most cases. If tuning such applications towards a same volume, as when following a specific contouring guideline or a study protocol, consistently defined volumes curated to this reference as basis for algorithm training can be expected to have a positive impact on performance.

## Declaration of Competing Interest

The authors declare the following financial interests/personal relationships which may be considered as potential competing interests: Niemelä J and Akram S are employed by the MVision AI company whose software tool is investigated in this study. Other authors have nothing to declare.
