# Supplementary material for: Autosegmentation based on different-sized training datasets of consistently-curated volumes and impact on rectal contours in prostate cancer radiation therapy
Source: Phys Imaging Radiat Oncol. 2022 May 5;22:67–72. doi: 10.1016/j.phro.2022.04.007 (PMC9092250; doi:10.1016/j.phro.2022.04.007)
Supplement: Supplementary Data 1 [file mmc1.docx]

**Supplementary material**

**Materials and Methods**

**The MVision autosegmentation tool**

The contouring is done in a web application that runs in the MVision Cloud environment where the user uploads DICOM-RT imaging information [1], with or without its own structure information. The files are processed in an anonymized format and returned with an RT Structure Set file including the AI-generated volumes (linked to the uploaded DICOM data) according to the user-selected prediction model for the body region in question. Details on time savings and consistency improvements when using the produced volumes by a previous version of the application (version 1.1) have recently been published [2]. For the male prediction model, as used in this study, computational time to delineate all pelvic structures is, based on these data, expected to be around 1.5-2 minutes per patient when the application is run on a virtual machine with V100 GPU. For the prediction of rectum volumes, *MVision_v1* has been trained on a dataset with CT scans from 891 patients and, although sourced from multiple clinics (n<40), data are curated by the company. Multiple annotators have been involved in curating the rectal volumes to cranially end at the angle where the rectum turns horizontally into the sigmoid colon and caudally to include the anal canal.

The MVision Segmentation Service algorithm is based on an encoder-decoder network architecture (Figure 1). It processes the given scans in thin overlapping sections. The encoders are based on SE-ResNeXt-50 with a 32x4d template model in line with Hu *et al.* [3] and the decoder consists of DenseNet modules in line with Huang *et al.* [4]. The feature maps of the n-th global encoder are summed with the feature maps of the (n-1)-th encoder to expand the available context in the axial direction. Skip-connections are used to gradually fuse information across multiple levels of the encoder and decoder. Multiple segmentation heads (small set of layers) are placed at the end of the decoder, with each head containing a list of non-overlapping structures. Risk of overfitting is reduced by using both spatial and intensity transformation data augmentation techniques. The spatial transforms include left-right flipping, jitter during cropping, elastic deformation, and resizing. The intensity transformations include random locally smooth noise, brightness change, gamma transform, gaussian noise, gaussian blur, and sharpening. The model is trained using the ADAM optimizer (a method for stochastic optimization) which minimizes a combination of Dice loss and weighted cross-entropy loss [5]. The learning rate is reduced when the validation error plateaus. The training is stopped when the learning rate falls below a set threshold.

**Results**

**Characteristics of different algorithm versions during re-training**

The output from the four new algorithm versions during re-training based on reference rectal volumes in Dataset 2 are presented in Supplementary Table 1. In the training subsets (n=32/64/128/256), predicted rectal volumes ranged from 71.5±24.2 cc to 75.3±30.1 cc with the larger values for volumes by *MVision_v32* and smaller for volumes by *MVision_v128*. Averaged mean doses for all algorithm versions were in the range 24.2-25.1 Gy (SD=6.4-6.5 Gy) and averaged maximum doses in the range 65.7-66.5 Gy (SD=7.2-7.7 Gy).

In the validation subset (n=34), for a same algorithm version, volumes were typically somewhat larger than in the training datasets (75.7-78.5 cc with SD: 22.1-24.0 cc; larger values for volumes by *MVision_v256* and smaller for volumes by *MVision_v32*) with somewhat lower averaged mean doses (23.9-24.6 Gy with SD: 6.7-7.2 Gy) and higher averaged maximum doses but smaller differences between versions (67.2-67.3 Gy with SD: 6.9-7.0 Gy).

In the small-scale testing subset (n=35), volumes were also somewhat larger than in the training datasets but in the similar range as in the validation dataset (73.6-78.9 cc with SD: 24.8-29.3 cc; larger values for volumes by *MVision_v64* and smaller values for volumes by *MVision_v32*). Averaged mean doses ranged between 23.5-24.3 Gy (SD: 5.4-5.7 Gy) whilst differences between averaged maximum doses were almost unnoticeable between algorithm versions (mean=65.0 Gy with SD: 8.0-8.1 Gy).

**References**

1. Law, M.Y. and B. Liu, *Informatics in radiology: DICOM-RT and its utilization in radiation therapy.* Radiographics, 2009. **29**(3): p. 655-67.

2. Kiljunen, T., et al., *A Deep Learning-Based Automated CT Segmentation of Prostate Cancer Anatomy for Radiation Therapy Planning-A Retrospective Multicenter Study.* Diagnostics (Basel), 2020. **10**(11).

3. Hu, J., L. Shen, and S. Gan, *Squeeze-and-Excitation Networks*, in *IEEE Conference on Computer Vision and Pattern Recognition (CVPR)*. 2018. p. 7132-7141.

4. Huang, G., et al., *Denslely Connected Convolutional Networks*, in *IEEE Conference of Computer Vision and Pattern Recognition (CVPR)*. 2017. p. 4700-4708.

5. Kingma, D.P. and J.L. Ba, *Adam: A method for stochastic optimization*, in *International Conference on Learning Representations (ICLR)*. 2015.

**Tables**

***Supplementary Table 1.*** *Volume and dose characteristics of rectal volumes as given by the output from the retrained versions of the MVision algorithm using four different training dataset sizes with curated rectal volumes selected from 325 patients (MVision data).*

| **Metric:** | **Volume** | **Mean dose** | **Max. dose** |
| --- | --- | --- | --- |
| **Group** | **cm^3^** | **Gy** | **Gy** |
| training, *n=32* | 75.3±30.1 | 24.2±6.5 | 65.7±7.7 |
| validation | 75.7±24.0 | 24.6±7.2 | 67.2±7.0 |
| testing | 73.6±24.8 | 24.3±5.4 | 65.0±8.0 |
| training, *n=64* | 73.3±27.3 | 25.1±6.4 | 66.2±7.5 |
| validation | 78.3±24.2 | 23.9±6.8 | 67.2±6.9 |
| testing | 78.9±29.3 | 23.6±5.6 | 65.0±8.1 |
| training, *n=128* | 71.5±24.2 | 25.0±6.2 | 66.5±7.2 |
| validation | 77.3±23.4 | 24.0±6.6 | 67.3±7.0 |
| testing | 77.8±27.8 | 23.5±5.7 | 65.0±8.0 |
| training, *n=256* | 72.7±23.1 | 24.6±6.5 | 65.9±7.8 |
| validation | 78.5±22.1 | 23.9±6.7 | 67.2±7.0 |
| testing | 78.5±27.1 | 23.5±5.2 | 65.0±8.0 |

*Subsets of validation/testing groups (n=34/35) randomly selected for each training data subset, each with unique data and not included in any other subset used for algorithm retraining.*

*Abbreviations: Gy = Gray, Max. = maximum.*
